# Multiple myeloma with esophageal involvement: A rare case

**DOI:** 10.1002/deo2.71

**Published:** 2021-11-02

**Authors:** Misako Tohata, Yorimasa Yamamoto, Hiroshi Harada, Erika Yoshida, Yuichi Takano, Kuniyo Gomi, Masatsugu Nagahama, Hiroshi Takahashi

**Affiliations:** ^1^ Department of Internal Medicine Division of Gastroenterology Showa University Fujigaoka Hospital Kanagawa Japan; ^2^ Department of Internal Medicine Division of Hematology Showa University Fujigaoka Hospital Kanagawa Japan

**Keywords:** bone marrow, esophagus, extramedullary lesion, multiple myeloma, plasma cells

## Abstract

A 77‐year‐old man presented with a spinal cord tumor at the cervical 7/thoracic 1 level and pain and weakness in the right hand. Blood tests revealed anemia, renal dysfunction, and hyperproteinemia. Immunoelectrophoresis revealed the M‐protein component of immunoglobulin G gamma globulin. Bone marrow aspirate contained an increased number of atypical plasma cells. He was diagnosed with symptomatic myeloma and treated with radiation therapy, chemotherapy, and extradural tumor resection. Upper gastrointestinal endoscopy, performed because of anemia progression, revealed a 5‐mm submucosal tumor‐like elevated lesion in the upper thoracic esophagus. On white light observation, the lesion appeared whitish with a central redness. Our patient was diagnosed with extramedullary multiple myeloma. Extramedullary lesions are rare in the gastrointestinal tract. To our knowledge, this case is the first of multiple myeloma with esophageal involvement.

## INTRODUCTION

Multiple myeloma is a disease in which neoplastic plasma cells proliferate mainly in the bone marrow and produce M‐protein, resulting in hematopoietic disorders, renal disorders, and osteolytic lesions. Approximately 13% of patients with multiple myeloma have extramedullary lesions at the time of diagnosis or during follow‐up,^1^, ^2^ primarily in the liver, spleen, kidneys, and lymph nodes. In the gastrointestinal (GI) tract, these lesions are rare. To our knowledge, this case is the first report of multiple myeloma with esophageal involvement.

## CASE REPORT

A 77‐year‐old man presented to our hospital with a spinal cord tumor and spinous process destruction at the cervical (C) 7/thoracic (T) 1 level. He had a more than 10‐year history of cervical spondylosis with ongoing treatment at an orthopedic clinic. He recently visited another hospital because of right‐hand pain and weakness. On presentation, blood tests indicated multiple myeloma, and he was admitted to the Department of Hematology at our hospital.

**FIGURE 1:**
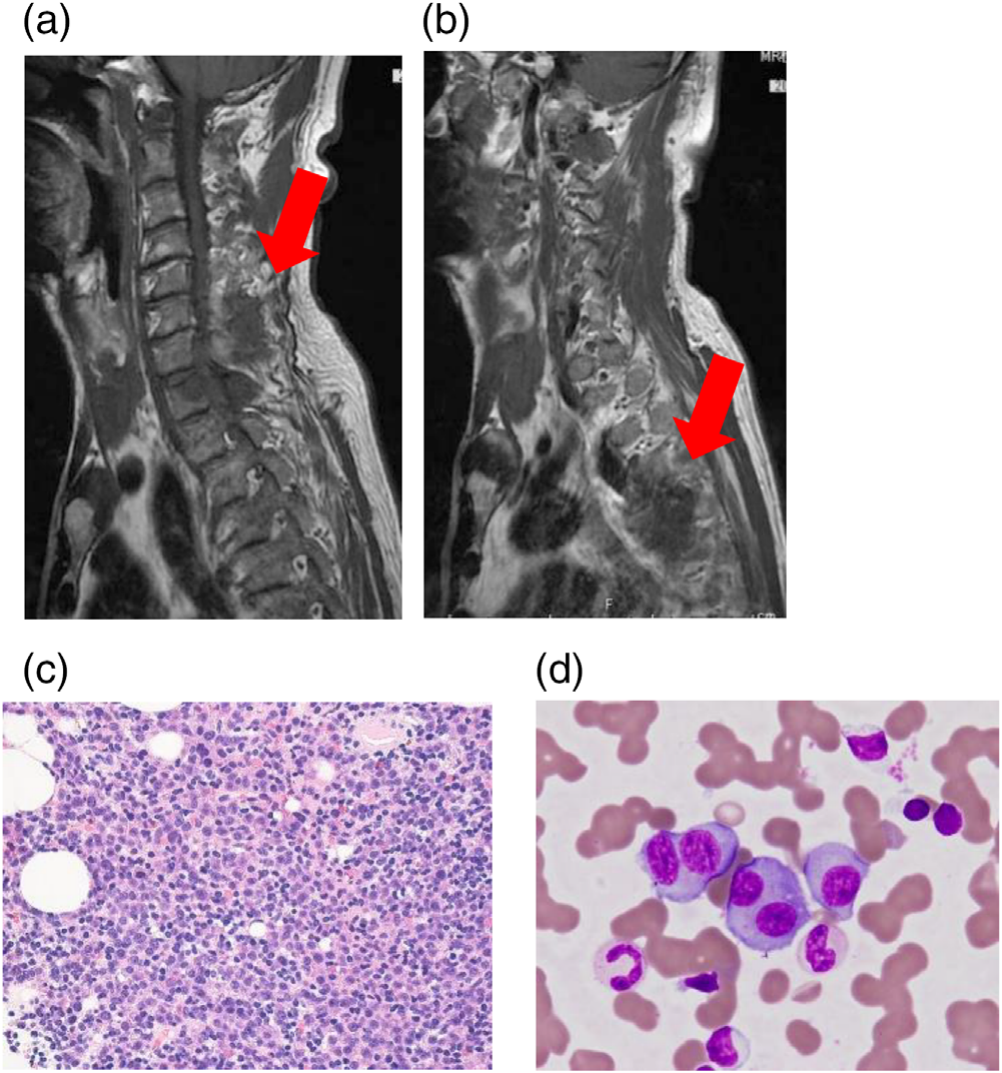
(a) Tumor shadow in the spinal cord at cervical 7/thoracic (T) 1 level (arrow). (b) Soft tissue mass shadow near the vertebral body at the T4/5 level (arrow). (c) Three lines of hematopoietic cells show a decrease, and a large number of atypical plasma cells characterized by perinuclear halo are observed by the hematoxylin‐eosin stain. (d) Plasma cells characterized by unevenly distributed nuclei and perinuclear halo are noted on Giemsa staining.

Laboratory findings on admission revealed anemia, renal dysfunction, and hyperproteinemia (Table 1). Immunoelectrophoresis showed the M‐protein component of immunoglobulin G gamma globulin. Cervical spine magnetic resonance imaging revealed soft tissue mass shadows suspected to be myeloma at C7/T1 and T4/5 levels (Figure 1a,b). Bone marrow findings (Figure 1c,d) showed plasma cells accounted for 31.9%. A large number of atypical plasma cells characterized by perinuclear halo were observed. Flow cytometry analysis of the bone marrow specimen demonstrated an increased number of cells that were positive for CD138, CD54, and the Cy lambda chain. Fluorescent in situ hybridization of the bone marrow showed chromosomal abnormalities with 13q deletion.

**TABLE 1:** Laboratory findings on admission

CBC	Biochemistry	
WBC 4100 /μl	TP 11.7 g/dl	IgA 30 mg/dl
Seg 55.0%	Alb 3.5 g/dl	IgM 4 mg/dl
Stab 1.0%	BUN 25 mg/dl	IgG 7516 mg/dl
Lympho 37.0%	UA 6.4 mg/dl	Free light chain
Mono 5.0%	CRE 1.06 mg/dl	Kappa 6 mg/L
Eosino 2.0%	Na 133 mEq/L	Lambda 254.0 mg/L
RBC 364 × 10^4^ /μl	Cl 99 mEq/L	β2MG‐s 6.2 mg/L
(Rouleaux formation)	K 4.4 mEq/L	Immunoelectrophoresis
Hb 12.7 g/dl	Ca 8.6 mg/dl	M component of IgG‐γ
Ht 37.6%	P 3.8 mg/dl	Urine
MCV 103 fl	GOT 19 U/L	β2MG‐u 2568 μg/L
MCH 34.9 pg	GPT 21 U/L	Bence‐Jones protein (−)
MCHC 33.8%	LDH 175 U/L	
Ret 0.7%	ALP 122 U/L	
Plt 19.8 × 10⁴/μl	CRP 0.56 mg/dl	

Symptomatic myeloma was diagnosed according to the International Myeloma Working Group diagnostic criteria.^3^ The serum β2 microglobulin level was >5.5 mg/L, indicating that the degree of serious illness was equivalent to stage III according to the International Staging System.

Radiation at C7/T8 at 30 Gy for 10 sessions was performed from Day 3 to Day 17 to improve neurologic symptoms. Treatment with bortezomib, melphalan, and prednisolone was also started on Day 7. Initially, the treatment course progressed without adverse effects; however, low back pain and nausea developed by Day 34, and neuropathy due to bortezomib was suspected. To reduce the dose of bortezomib as a key treatment drug, we changed the therapy to bortezomib plus dexamethasone starting on Day 43. The melphalan was discontinued because it is not included in this regimen.

On Day 68 day, lower limb weakness developed, and thoracic spine magnetic resonance imaging showed spinal cord compression due to an epidural tumor at the T10–11 level, for which the patient underwent laminectomy and partial tumor resection. Pathologic findings revealed that the tumor was a plasmacytoma. Radiation therapy (T10/sacral (S) 1 at 30 Gy for 10 sessions) was performed again on Days 84–98 to relieve the pain caused by the mass.

On Day 103, therapy with Revlimid plus dexamethasone was started. Anemia gradually progressed, and the hemoglobin concentration decreased to 6 g/dl.

**FIGURE 2:**
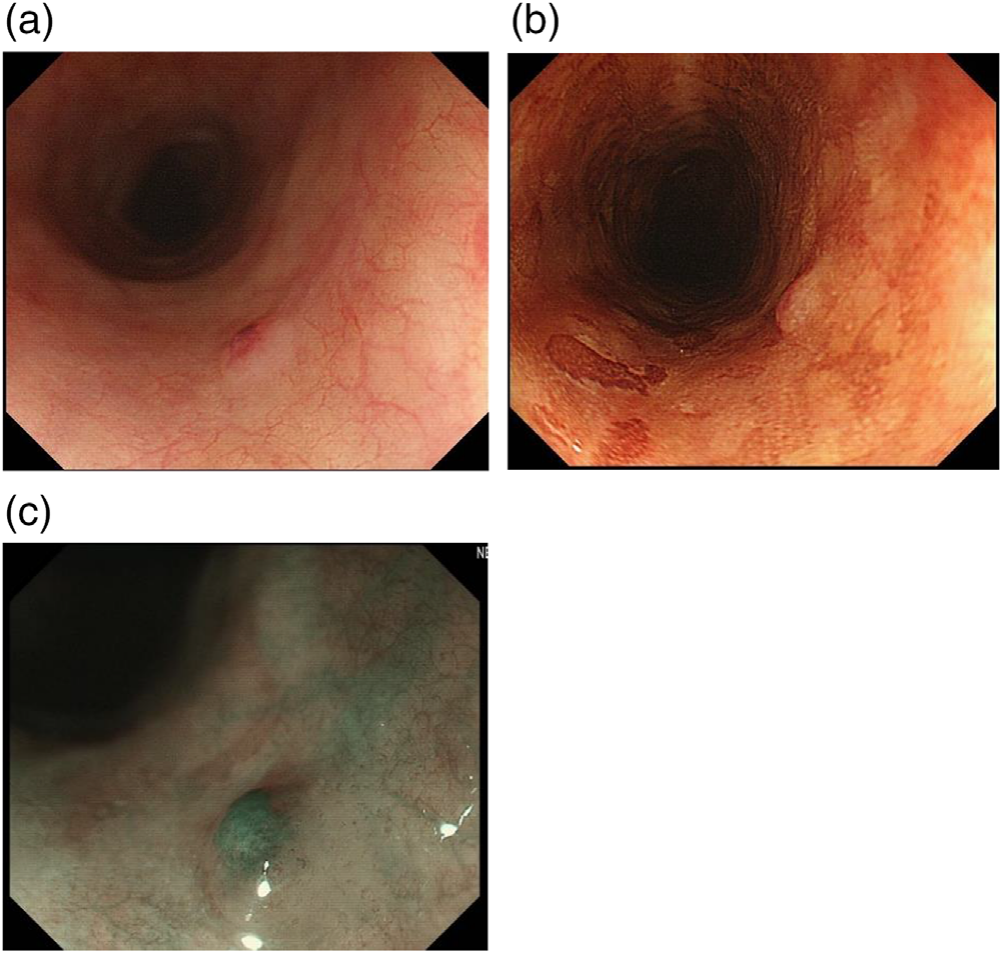
(a) Upper gastrointestinal endoscopy shows a 5‐mm submucosal tumor‐like elevated lesion in the upper thoracic esophagus. On white light observation, the lesion appeared whitish with a central redness. (b) After iodine staining, the lesions are only faintly stained. (c) In narrow‐band imaging, the central part of the elevated lesion, showing redness with white light observation, appeared green and showed an unclear boundary of the elevation, with small submucosal tumor‐like findings.

**FIGURE 3:**
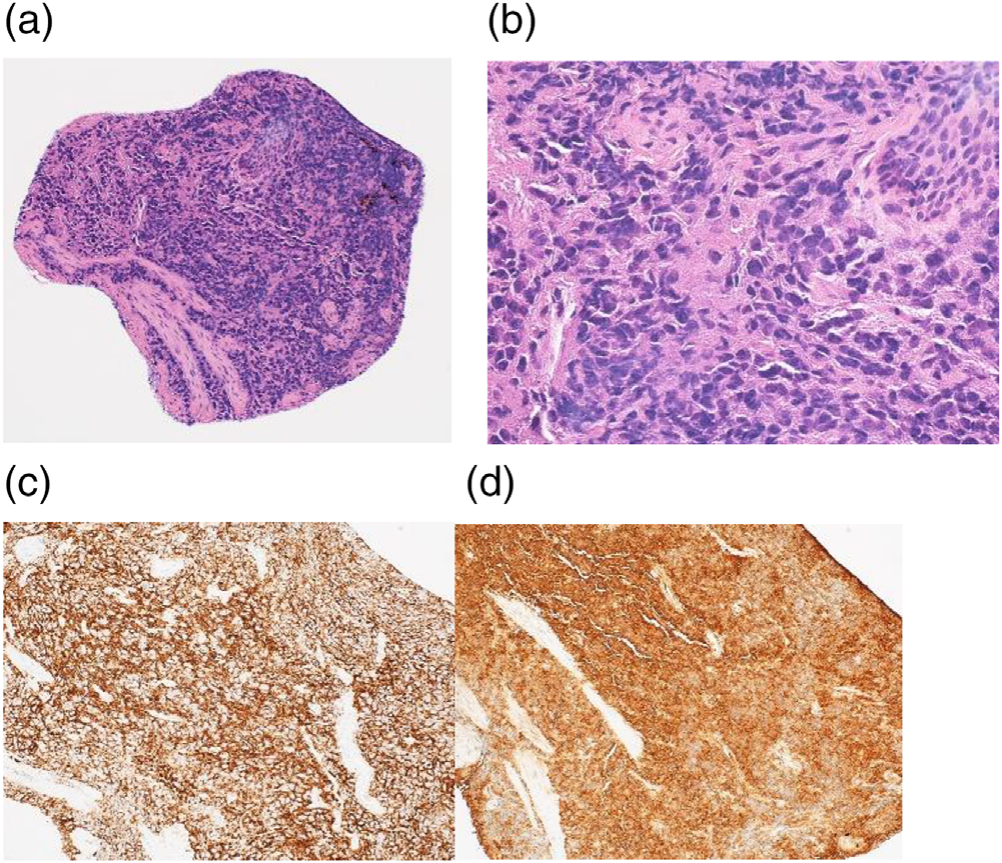
(a, b) Histologic examination with hematoxylin‐eosin stain confirmed that plasma cells with some variants developed in the mucosal layer. (c) Immunostaining shows positive CD138. (d) Immunostaining shows a positive lambda chain.

On Day 114, upper GI endoscopy revealed a 5‐mm submucosal tumor‐like elevated lesion in the upper thoracic esophagus. On white light observation, the lesion appeared whitish with a central redness (Figure 2a). On iodine staining, the lesions were only faintly stained (Figure 2b). On narrow‐band imaging, the central part of an elevated lesion showed redness with white light observation, appeared green with an unclear boundary of elevation, and showed small submucosal tumor‐like findings (Figure 2c). Histologic examination with hematoxylin‐eosin stain confirmed that plasma cells with some variants in the mucosal layer (Figure 3a,b). Immunostaining demonstrated positive CD138 and lambda chain (Figure 3c,d). Histologic examination of the biopsy samples indicated myeloma cell infiltration.

It is necessary to distinguish between primary and extramedullary lesions of multiple myeloma in plasmacytoma in the GI tract; however, it has been reported that extramedullary lesions often occur in stage‐3 cancer.^1^ This case was diagnosed as an extramedullary lesion because it was a stage 3 advanced multiple myeloma and the esophageal lesion was a small mass.

After this diagnosis, on Day 121, the second course of Revlimid plus dexamethasone therapy was started. The right‐back pain appeared, and computed tomography showed a mass around the kidney and lymphadenopathy at the bifurcation of the trachea. Chemotherapy was ineffective, and re‐irradiation (T5–11 at 30 Gy for 10 sessions) was performed, but no sufficient effect was obtained. A blood test showed an increase in lactate dehydrogenase and plasma cells in the peripheral blood. The patient's general condition gradually deteriorated, and he died about 5 months after the diagnosis.

## DISCUSSION

Myeloma is a disease in which plasma cells, which are the final differentiation stage of B lymphoid cells, show neoplastic proliferation. The disease is classified into five types based on the infiltration mode: multiple myeloma, solitary myeloma, extramedullary plasmacytoma, and plasma cell leukemia. Of these types, multiple myeloma is the most common.

Extramedullary lesions of multiple myeloma are reported to occur in approximately 13% of patients, 7% at diagnosis and 6% during the course of the disease,^1^ chiefly in the liver, spleen, kidneys, and lymph nodes. These lesions are rare in the GI tract, reportedly occurring in only 24 of 2584 patients (0.9%).^4^ These 24 cases presented throughout the GI tract as follows: 11 liver, eight pancreas, four stomach, two duodenum, one ileum, three colon, and one rectum case (including duplicate examples).

Most stomach cases were submucosal tumor‐like^5^, ^6^ and advanced cancer‐like large lesions, but also erosions and reddish, small, flat lesions.^7^ Therefore, the morphology is diverse. In the stomach, multiple lesions have been reported, but many are single lesions. Previous reports have also described single lesions in the small and large intestines. The reason is unclear, but single lesions are more common in the GI tract, and our case of an esophageal lesion was also single.^5^, ^6^, ^8^

In the cases of small and large intestine lesions, abdominal pain and GI bleeding are common,^8^ and ileus and perforation due to a giant mass and stenosis have been reported. In addition, Mochizuki et al.^6^ reported a case of a gastric lesion and rectal lesion; therefore, it is considered desirable to examine the entire GI tract when the extramedullary lesion is found in the GI tract. In this case, lower GI endoscopy was not performed when the anemia progressed, considering the patient's general condition. Computed tomography showed no obvious lesions in the small or large intestine, and no bleeding or stenosis, but did show tumor infiltration around the kidney.

In this case, esophagus involvement by multiple myeloma, submucosal tumor, and cancer was considered as differential diagnoses. However, diagnosis based on endoscopic findings alone was difficult because the typical findings of esophagus involvement by multiple myeloma were not reported. We think that endoscopic ultrasonography can be used for detailed observation. However, in this case, we performed only a biopsy because the patient's condition was poor and we were focused on investigating the source of bleeding that was causing the anemia.

Several other reports have documented bleeding and perforation in the stomach and intestinal lesions, but our patient did not present with these signs.^5^, ^8^ Of note, symptoms such as stenosis and bleeding may occur as lesions grow.

Extramedullary lesions in the esophagus are extremely rare and have not been reported to date. The mechanism for extramedullary lesions is attributed to blood and lymphatic vessels. In this case, the cervical and thoracic spine myeloma were irradiated and partially resected. It is possible that myeloma cells settled in the esophagus through the vessels due to therapeutic invasion. However, even after confirming in past reports, the relationship between extramedullary lesions of the GI tract and the myeloma site remains unclear. Regarding the formation of extramedullary lesions, fulminant multiple myeloma^9^ with extramedullary lesions and chromosomal abnormalities, including 13q deletion, have been documented in case reports with multiple extramedullary lesions.^6^ Chromosomal abnormalities of 13q deletion have been reported as poor prognostic factors. This chromosomal abnormality was also present in this case and may be a factor that caused extramedullary lesions, although the esophagus is an extremely rare sight. In conclusion, GI extramedullary lesions in multiple myeloma are rare, but we should consider the possibility of extramedullary lesions, including the esophagus, for cases of stage‐3 cancer.

## CONFLICT OF INTEREST

The authors declare that they have no conflict of interest.

## FUNDING INFORMATION

None.

## ETHICS STATEMENT

All procedures followed have been performed in accordance with the ethical standards laid down Declaration of Helsinki and its later amendments. Since it is difficult to obtain written consent from the person regarding the presentation, the ethics committee of this hospital approved the presentation.
